# Strengthening primary care gatekeeping for healthy aging in China: family doctor contract service and multidimensional health outcomes among older adults

**DOI:** 10.3389/fpubh.2026.1875855

**Published:** 2026-08-18

**Authors:** Cangcang Jia, Zhengyang Wang, Ling Zhang, Jin Wang

**Affiliations:** 1 School of Health Policy & Management, Nanjing Medical University, Nanjing, China; 2 School of Government, Nanjing University, Nanjing, China; 3 Nanjing Drum Tower Hospital, Nanjing University, Nanjing, China

**Keywords:** family doctor contract service, health equity, health outcomes, life satisfaction, older adults

## Abstract

**Objective:**

Expanding access to community-based primary care is central to addressing the health challenges posed by population aging in China. This study examines associations between Family Doctor Contract Service (FDCS) participation and multidimensional health outcomes among community-dwelling older adults.

**Methods:**

Using nationally representative data from the 2023 China Longitudinal Aging Social Survey (*N* = 11,455), we assessed three health dimensions: self-rated health, physical discomfort, and life satisfaction. OLS regression provided a transparent baseline of adjusted associations, ordered probit and logit models served as robustness checks for ordinal outcomes, and propensity score matching was used as a covariate-balance adjustment tool for observable selection into FDCS participation. Subgroup analyses examined heterogeneity by urban or rural residence and education level.

**Results:**

FDCS participation was significantly associated with more favorable observed outcomes across all three health dimensions. Rural and less-educated older adults showed stronger associations with lower reported physical discomfort and higher life satisfaction, whereas urban and higher-educated older adults showed stronger associations with self-rated health appraisals. This pattern suggests heterogeneous associations between FDCS participation and health outcomes across population groups.

**Conclusion:**

The findings suggest that reported FDCS participation is associated with multiple dimensions of older adults’ health, with heterogeneous patterns across residence and education groups. Differentiated service design may help align community-based primary care with the observed needs of different older adult subpopulations. All findings reflect adjusted associations from a cross-sectional observational study and should not be interpreted as causal estimates.

## 1 Introduction

Population aging represents a defining global challenge, with China experiencing particularly rapid demographic transition (1–3). By the end of 2024, individuals aged 60 and above constituted 22.0% of China’s population, totaling 310 million persons, while those aged 65 and above reached 220 million, accounting for 15.8% of the population (4). This demographic shift fundamentally restructures national health systems and healthcare demand structures, as older adults experience heightened vulnerability to chronic diseases, functional limitations, and multidimensional health challenges (5). The rapid expansion of the older adult population places unprecedented pressure on healthcare delivery systems, manifesting in increased demand for primary care services, chronic disease management, and continuous health monitoring. Traditional hospital-centric healthcare models prove inadequate for meeting the complex, long-term care needs of aging populations, necessitating a fundamental shift toward strengthened community-based primary care capacity. The healthcare system must transition from episodic, acute care delivery to continuous, preventive care coordination tailored to the multidimensional health needs of older adults (6–8).

In response to these mounting challenges, China launched the Family Doctor Contract Service (FDCS) program in 2016 as a cornerstone initiative within primary healthcare reform (9–11). The program delivers comprehensive, continuous, and community-based care through multidisciplinary teams comprising general practitioners, public health physicians, and nurses, with core services encompassing routine health examinations, chronic disease management, health education, and personalized care coordination (12, 13). FDCS represents a strategic effort to reorient healthcare delivery from hospital-centric, episodic treatment toward continuous primary care tailored to the long-term needs of an aging population (14, 15).

A growing body of research has examined FDCS associations with health outcomes among older adults, with evidence clustering around three domains. In the domain of clinical disease management, studies consistently report positive associations between FDCS participation and chronic disease control and medication adherence among older adults with hypertension and diabetes (15–21). In the domain of healthcare use and service quality, research links FDCS participation with better perceived primary care quality (22) and social adaptability (23). A smaller strand of work has extended to broader subjective well-being outcomes. Despite this accumulating evidence, two limitations constrain current understanding. First, existing studies largely examine a single health dimension, leaving unclear whether FDCS associations differ across cognitive, perceptual, and affective domains of health (24–26). Second, no study has systematically assessed whether these associations are heterogeneous across socioeconomic subgroups, a question with direct implications for evaluating FDCS as an equity-promoting intervention.

To address these gaps, we draw on Andersen’s Behavioral Model of Health Services Use (27) and the social determinants of health perspective (28) to develop an integrative dual-pattern framework. The first interpretation is accessibility-oriented. For rural and less-educated older adults facing greater barriers to care, FDCS participation may be associated with lower travel, information, and coordination burdens, corresponding to relatively larger associations with perceptual and affective outcomes such as physical discomfort and life satisfaction. The second interpretation concerns health literacy and self-management. For urban and higher-educated older adults with stronger baseline access, regular FDCS consultations may be associated with greater health awareness and preventive behavior, corresponding to larger associations with self-rated health. This framework generates an explicit expectation of heterogeneous association patterns that distinguishes the present study from prior single-outcome research. We emphasize that this framework was not pre-registered before data analysis. It was instead developed by synthesizing these established theories with the empirical patterns observed in our data. We therefore distinguish throughout between our pre-study expectation that the associations between FDCS and health would differ across socioeconomic groups, and the specific dual-pattern interpretation, which is an integrative and theory-informed account of the observed heterogeneity rather than a confirmatory test of pre-specified hypotheses.

Using nationally representative data from the 2023 China Longitudinal Aging Social Survey (CLASS) comprising 11,455 older adults, this study addresses two research questions. First, is FDCS participation associated with better outcomes across multiple health dimensions including self-rated health, physical discomfort, and life satisfaction among older adults in China? Second, do the patterns and magnitudes of these associations vary across population subgroups defined by residence and education, consistent with the dual-pattern framework developed above?

## 2 Methods

### 2.1 Data and sampling

This study used data from the 2023 wave of the China Longitudinal Aging Social Survey (CLASS), a nationally representative survey conducted by the Center for Population and Development Studies and the Institute of Gerontology at Renmin University of China (RUC). The CLASS employs a multistage stratified probability sampling design covering 28 provinces, autonomous regions, and municipalities across China. The survey recruited older adults aged 60 and above and was conducted at respondents’ homes through face-to-face interviews. The study was approved by the Ethics Committee of Renmin University of China. The 2023 wave contained 11,739 participants from over 450 village/community-level units and 150 counties/districts. The collected information includes demographic and socioeconomic characteristics, health status, healthcare use, family relationships, and social support networks. We used the 2023 wave because it was the first CLASS wave to include comprehensive questions on reported family doctor contract service (FDCS) participation among older adults. This study examines the associations between reported FDCS participation and multidimensional health outcomes among older adults. We excluded respondents who did not answer questions related to family doctor contract service and those with missing data on key outcome variables. After applying these exclusion criteria, the final analytical sample comprised 11,455 older adults.

### 2.2 Variables definition

#### 2.2.1 The dependent variable

Following established approaches in gerontological health research, this study employed a multidimensional framework to assess older adults’ health outcomes, encompassing physical, mental, and subjective well-being indicators. Self-rated health served as the primary outcome measure, assessed through the question “How do you feel about your current physical health status?” with responses coded on a five-point scale from 1 “very unhealthy” to 5 “very healthy.” Self-rated health (SRH) is conceptualized as a cognitive and global appraisal of current health status, not an objective clinical diagnosis. SRH may overestimate or underestimate objective health status and may be influenced by expectations, adaptation, and the tendency to equate absence of diagnosed illness with health. SRH is nonetheless retained because a large validation literature shows its robust predictive validity for mortality and functional decline among older adults (29). Physical discomfort was measured as a binary variable based on the question “Did you experience any physical pain in the past month?” coded as 0 for “no” and 1 for “yes,” conceptualized as a recent perceptual symptom experience. Life satisfaction was evaluated using a five-point scale ranging from 1 “very dissatisfied” to 5 “very satisfied,” treated as an affective-evaluative assessment of overall well-being. Physical discomfort and self-rated health are related but not interchangeable. Physical discomfort captures a recent perceptual symptom experience, whereas SRH additionally incorporates chronic disease knowledge, functional expectations, age-related social comparison, and psychological adaptation. The partial divergence of results across the two outcomes indicates empirically that they measure distinct constructs. In the Chinese older adult context, SRH may partly reflect the culturally common equation of absence of diagnosed illness with being healthy. Physical discomfort and life satisfaction were therefore included as co-primary outcomes to capture experiential and affective dimensions of health that are less susceptible to this reporting tendency.

#### 2.2.2 Independent variable

FDCS was measured through the question “Have you used contracted family doctor services in the past 12 months?” In this study, FDCS participation refers to self-reported use of contracted family doctor services at least once during the past 12 months, reflecting actual engagement with the program rather than formal registration or contract signing alone. This binary variable captures reported FDCS participation in the past 12 months rather than service intensity, frequency, duration, continuity, or quality, and it does not differentiate these dimensions of service receipt among participants. Participation in China’s community-based primary healthcare initiative aligns with the broader concept of community-based health services that have been associated with better health outcomes among older adults with chronic diseases. Throughout the manuscript, we therefore use the term “FDCS participation” to denote reported engagement with contracted family doctor services, while avoiding the stronger implication that the measure captures the actual intensity or quality of healthcare use. This operationalization is consistent with international definitions of community-based health services that emphasize regular home visits, care coordination, and health promotion activities delivered within familiar community settings.

#### 2.2.3 Control variables

Covariates are selected *a priori* based on Andersen’s Behavioral Model of Health Services Use and the social determinants of health perspective. Predisposing characteristics include age, gender, and marital status (25); enabling resources include education, residence, and personal income (30); need factors include chronic disease status (31); and health-behavioral and digital-access factors include smoking, physical exercise, and internet use (32). These variables are theoretically relevant because they may simultaneously influence the probability of FDCS participation and older adults’ health outcomes; accordingly, they are included in both regression adjustment and propensity score estimation. Age is treated as a continuous variable measured in years and subsequently categorized following World Health Organization guidelines and gerontological literature into two groups: young-old adults (aged 60–79 years, *N* = 4,655) and oldest-old adults (aged 80 years and above, *N* = 6,800). Gender is coded as a binary variable with male = 1 and female = 0. Marital status distinguishes between currently married with spouse present (coded as 1) and all other marital statuses including widowed, divorced, and never married (coded as 0). Educational background is operationalized through dummy variables representing primary school, middle school, and high school or above education levels, using illiteracy as the baseline category. Residential location is captured as a binary variable distinguishing urban (coded as 1) from rural areas (coded as 0). Personal annual income is measured continuously and subjected to logarithmic transformation to normalize its skewed distribution for analytical purposes. Chronic disease burden is assessed through dummy variables indicating single chronic disease and multiple chronic diseases, with absence of chronic conditions as the reference group. Smoking behavior is coded as a binary indicator where 1 represents any smoking history and 0 indicates never smoking. Regular physical exercise participation is measured as a binary variable (1 = regular exercise, 0 = no regular exercise). Internet use is similarly coded as a binary measure (1 = regular internet use, 0 = no internet use). Table 1 presents the descriptive statistics for all variables included in the analysis.

**Table 1:** Descriptive statistics of sample characteristics.

Variables	Total sample (*N* = 11,455)		FDCS participants (*N* = 2,170)		Non-participants (*N* = 9,285)		*p*
	Mean/n	SD/%	Mean/n	SD/%	Mean/n	SD/%	
Self-rated health							0.000
Very unhealthy	93	0.81	10	0.46	83	0.89	
Relatively unhealthy	814	7.11	133	6.13	681	7.33	
Average	3,986	34.80	702	32.35	3,284	35.37	
Relatively healthy	5,424	47.35	1,199	55.25	4,225	45.50	
Very healthy	1,138	9.93	126	5.81	1,012	10.90	
Physical discomfort							0.0000
No	7,732	67.50	1,548	71.34	6,184	66.60	
Yes	3,723	32.50	622	28.66	3,101	33.40	
Life satisfaction							0.000
Very dissatisfied	59	0.52	7	0.32	52	0.56	
Relatively dissatisfied	416	3.63	74	3.41	342	3.68	
Average	2,684	23.43	348	16.04	2,336	25.16	
Relatively satisfied	6,358	55.50	1,396	64.33	4,962	53.44	
Very satisfied	1,938	16.92	345	15.90	1,593	17.16	
Age							0.5226
Young-old	4,655	40.64	895	41.24	3,760	40.50	
Oldest-old	6,800	59.36	1,275	58.76	5,525	59.50	
Gender							0.3019
Female	5,525	48.23	1,025	47.24	4,500	48.47	
Male	5,930	51.77	1,145	52.76	4,785	51.53	
Marriage							0.0011
Other	1,938	16.92	316	14.56	1,622	17.47	
Married	9,517	83.08	1,854	85.44	7,663	82.53	
Education							0.000
Illiteracy	2,299	20.07	469	21.61	1,830	19.71	
Primary school	4,256	37.15	633	29.17	3,623	39.02	
Middle school	3,196	27.90	621	28.62	2,575	27.73	
High school and above	1,704	14.88	447	20.60	1,257	13.54	
Residence							0.0000
Rural	5,626	49.11	866	39.91	4,760	51.27	
Urban	5,829	50.89	1,304	60.09	4,525	48.73	
Chronic disease							0.000
No chronic disease	2,435	21.26	325	14.98	2,110	22.72	
Single chronic disease	3,305	28.85	523	24.10	2,782	29.96	
Multiple chronic diseases	5,715	49.89	1,322	60.92	4,393	47.31	
Smoking							0.5429
No	8,328	72.70	1,589	73.23	6,739	72.58	
Yes	3,127	27.30	581	26.77	2,546	27.42	
Internet use							0.0000
No	5,338	46.60	835	38.48	4,503	48.50	
Yes	6,117	53.40	1,335	61.52	4,782	51.50	
Physical exercise							0.0002
No	6,340	55.35	1,124	51.80	5,216	56.18	
Yes	5,115	44.65	1,046	48.20	4,069	43.82	
Personal annual income (log)	10.1713	2.2568	10.1138	1.8509	10.1848	2.3414	0.000

### 2.3 Statistical analyses

Ordinary least squares (OLS) is used as a transparent baseline because its coefficients can be interpreted directly as adjusted mean differences, and prior studies of ordered subjective outcomes have shown that substantive conclusions are often similar when OLS results are corroborated by ordered response models (33, 34). We do not interpret OLS as resolving the ordinal-scale issue; therefore, ordered probit and ordered logit models are reported as scale-appropriate robustness checks. The fundamental regression specification is expressed as:


$$\text{Healt}h_{i}=\alpha +\beta_{1}\mathrm{FDC}S_{i}+\beta_{2}X_{i}^{’}+\varepsilon_{i}$$ (1)

In Equation 1, Healthi represents the dependent variable reflecting various health outcomes of older adult i, including self-rated health, physical discomfort, life satisfaction, health status compared to peers, and health changes. The key independent variable FDCSi indicates whether older adult i reported FDCS participation. Xi’ represents the vector of control variables encompassing demographic, socioeconomic, and health-related characteristics. The coefficient beta1 captures the estimated associations between FDCS participation and health outcomes, with its magnitude and statistical significance indicating the adjusted relationship between reported participation and health status among older adults. The term epsilon_i represents the random error term.

The empirical strategy follows a three-level structure. OLS regression provides a transparent baseline of adjusted associations; ordered probit and logit models test whether conclusions hold under ordinal scaling assumptions; and propensity score matching is used as a covariate-balance adjustment tool for observable selection into FDCS participation, serving as a matched-design complement to regression adjustment. Propensity score matching (PSM) is implemented using four algorithms, namely k-nearest neighbor (k = 4), radius matching (caliper = 0.01), kernel matching, and Mahalanobis distance matching. Propensity scores are estimated via logistic regression including demographic (age, gender, marital status), socioeconomic (education, residence, income), behavioral (smoking, exercise, internet use), and health (chronic diseases) covariates. Balance diagnostics confirm successful covariate balancing using standardized mean differences.

Heterogeneity analysis examines differential associations by residence (urban vs. rural) and education (primary school and below vs. middle school and above). Residence and education were dichotomized *a priori* because these divisions correspond to major structural inequalities in China’s primary care access and older cohorts’ educational opportunities, while also preserving sufficient sample size and common support for subgroup-specific matching. As documented in Table 1, substantial covariate imbalance exists between FDCS participants and non-participants in the full sample, and similar imbalances persist within each subgroup stratum. Urban FDCS participants differ systematically from urban non-participants on education, chronic disease burden, and socioeconomic characteristics, as do rural participants from rural non-participants. A stratified OLS comparison would therefore conflate differential selection with subgroup-specific associations. Heterogeneity analysis is accordingly conducted within the PSM framework, with separate matching procedures applied within each stratum to achieve covariate balance before estimating matched association differences. This ensures that subgroup-specific estimates are comparable across strata and methodologically consistent with the overall association-analysis strategy. As robustness checks on the binary groupings, we re-estimate the subgroup models using a four-level education classification covering illiterate, primary school, middle school, and high school or above, and a migration-sensitive residence classification that distinguishes non-migrant rural and urban older adults from rural-to-urban and urban-to-rural migrants, with full results reported in the Discussion and in Supplementary Tables S1, S2. All statistical analyses were performed using Stata SE 18.0 software. Statistical significance was set at *p* < 0.05, with 95% confidence intervals reported for all estimates.

## 3 Results

### 3.1 The characteristics of the study samples

Table 1 summarizes the baseline characteristics of the analytical sample, stratified by reported FDCS participation. Based on the 2023 CLASS data, the sample included 11,455 adults aged 60 years or older, with 2,170 (18.9%) reporting FDCS participation. Significant differences were observed across groups: participants showed a lower prevalence of physical discomfort (28.66% vs. 33.40%, *p* < 0.001). Demographically, participants exhibited higher educational attainment (20.60% vs. 13.54% with high school or above, *p* < 0.001), greater urban residence (60.09% vs. 48.73%, *p* < 0.001), and a higher burden of multiple chronic conditions (60.92% vs. 47.31%, *p* < 0.001). Internet use was more common among participants (61.52% vs. 51.50%, *p* < 0.001), and physical exercise participation was also higher (48.20% vs. 43.82%, *p* < 0.001), whereas age and gender distributions were balanced (*p* > 0.05). These patterns reflect potential selection patterns in FDCS participation, with participants characterized by better socioeconomic resources yet poorer baseline health status, motivating the use of propensity score matching as a covariate-balance adjustment tool.

### 3.2 Associations of family doctor contract service participation with health outcomes among older adults

**Figure 1:**
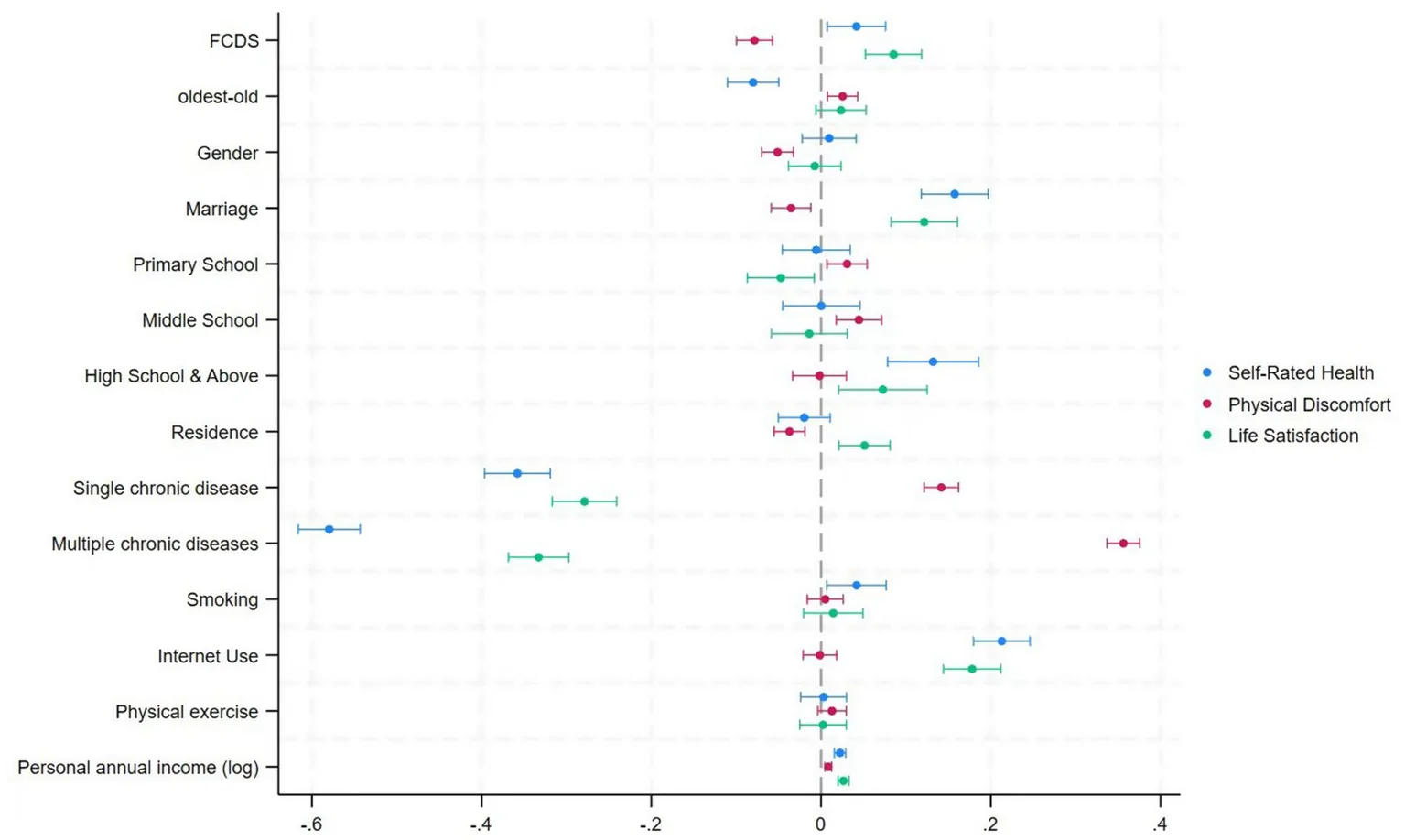
Forest plot of covariate associations with health outcomes from baseline OLS regression.

The baseline OLS regression analysis controlled for a comprehensive set of demographics, socioeconomic, and behavioral covariates to examine the association between FDCS participation and health outcomes among older adults. As detailed in Table 2, reported FDCS participation showed statistically significant positive associations across all three health dimensions. Specifically, FDCS participation was associated with higher self-rated health (beta = 0.0417, *p* < 0.05), lower reported physical discomfort (beta = −0.0785, *p* < 0.001), and greater life satisfaction (beta = 0.0854, *p* < 0.001). Figure 1 provides a comprehensive visualization of the estimated associations of all covariates with health outcomes, facilitating direct comparison of association magnitudes and precision across different predictors. Among control variables, chronic disease burden exhibited the strongest associations with health outcomes, whereby multiple chronic conditions were associated with lower self-rated health (beta = −0.5796, *p* < 0.001), lower life satisfaction (beta = −0.3328, *p* < 0.001), and higher physical discomfort (beta = 0.3563, *p* < 0.001). Internet use was associated with higher self-rated health (beta = 0.2129, *p* < 0.001) and life satisfaction (beta = 0.1780, *p* < 0.001), whereas marital status showed significant positive associations with better outcomes across all health dimensions. The models explained 15.66, 10.53, and 8.73% of the variance in self-rated health, physical discomfort, and life satisfaction respectively, indicating reasonable explanatory power for the analytical framework employed.

**Table 2:** OLS estimates of associations between FDCS and health outcomes among older adults.

Variables	(1)	(2)	(3)
	Self-rated health	Physical discomfort	Life satisfaction
FCDS	0.0417^**^	−0.0785^***^	0.0854^***^
	[0.0073,0.0761]	[−0.0996,−0.0573]	[0.0523,0.1186]
Oldest-old	−0.0800^***^	0.0254^***^	0.0235
	[−0.1102,−0.0498]	[0.0075,0.0433]	[−0.0061,0.0531]
Gender	0.0096	−0.0513^***^	−0.0074
	[−0.0222,0.0413]	[−0.0701,−0.0326]	[−0.0385,0.0237]
Marriage	0.1575^***^	−0.0353^***^	0.1216^***^
	[0.1181,0.1968]	[−0.0587,−0.0120]	[0.0825,0.1607]
Primary school	−0.0057	0.0307^**^	−0.0474^**^
	[−0.0457,0.0343]	[0.0070,0.0543]	[−0.0869,−0.0080]
Middle school	0.0003	0.0446^***^	−0.0138
	[−0.0452,0.0459]	[0.0179,0.0714]	[−0.0585,0.0310]
High school & above	0.1321^***^	−0.0018	0.0729^***^
	[0.0785,0.1857]	[−0.0336,0.0300]	[0.0207,0.1250]
Residence	−0.0198	−0.0372^***^	0.0512^***^
	[−0.0503,0.0107]	[−0.0553,−0.0191]	[0.0211,0.0814]
Single chronic disease	−0.3578^***^	0.1418^***^	−0.2788^***^
	[−0.3964,−0.3192]	[0.1215,0.1621]	[−0.3168,−0.2408]
Multiple chronic diseases	−0.5796^***^	0.3563^***^	−0.3328^***^
	[−0.6161,−0.5431]	[0.3370,0.3757]	[−0.3683,−0.2973]
Smoking	0.0418^**^	0.0050	0.0144
	[0.0067,0.0768]	[−0.0162,0.0262]	[−0.0206,0.0494]
Internet use	0.2129^***^	−0.0014	0.1780^***^
	[0.1797,0.2462]	[−0.0211,0.0183]	[0.1443,0.2118]
Physical exercise	0.0030	0.0129	0.0024
	[−0.0241,0.0300]	[−0.0039,0.0296]	[−0.0249,0.0297]
Personal annual income (log)	0.0223^***^	0.0085^***^	0.0265^***^
	[0.0156,0.0290]	[0.0046,0.0124]	[0.0201,0.0329]
_cons	3.5202^***^	0.0644^**^	3.5814^***^
	[3.4350,3.6054]	[0.0151,0.1138]	[3.4977,3.6650]
*N*	11,455	11,455	11,455
*R* ^2^	0.1566	0.1053	0.0873
adj. *R*^2^	0.1556	0.1042	0.0862

95% confidence intervals in brackets. ^*^p < 0.1, ^**^p < 0.05, ^***^p < 0.01.

### 3.3 Robustness test

#### 3.3.1 Alternative model specifications

Given the non-continuous nature of our dependent variables, which include ordinal and binary measures, we re-estimated baseline models using ordered probit and ordered logit specifications to address potential biases from OLS assumptions. Table 3 presents results from both alternative specifications, showing substantial consistency with baseline estimates and supporting the robustness of our main association patterns. FDCS participation remains significantly associated with lower reported physical discomfort (Oprobit: beta = −0.2258, *p* < 0.01; Ologit: beta = −0.3932, *p* < 0.01) and greater life satisfaction (Oprobit: beta = 0.1238, *p* < 0.01; Ologit: beta = 0.2225, *p* < 0.01) across all specifications. For self-rated health, the association remains statistically significant in the Ologit model (beta = 0.1019, *p* < 0.05) and shows a consistent positive direction in the Oprobit model (beta = 0.0463, *p* < 0.1), though the latter does not reach conventional statistical significance. These results provide support for positive associations between FDCS participation and multiple dimensions of older adult health, with the most robust evidence for lower reported physical discomfort and greater life satisfaction.

**Table 3:** Robustness check: replacing OLS with ordered probit and ordered logit models.

Variables	Oprobit model			Ologit model		
	Self-rated health	Physical discomfort	Life satisfaction	Self-rated health	Physical discomfort	Life satisfaction
FCDS	0.0463^*^	−0.2258^***^	0.1238^***^	0.1019^**^	−0.3932^***^	0.2225^***^
	[−0.0052,0.0978]	[−0.2922,−0.1594]	[0.0727,0.1749]	[0.0115,0.1923]	[−0.5047,−0.2818]	[0.1345,0.3105]
Oldest-old	−0.1214^***^	0.0748^***^	0.0390^*^	−0.2204^***^	0.1315^***^	0.0572
	[−0.1671,−0.0756]	[0.0194,0.1302]	[−0.0065,0.0845]	[−0.3014,−0.1394]	[0.0391,0.2239]	[−0.0230,0.1373]
Gender	0.0104	−0.1600^***^	−0.0128	0.0232	−0.2655^***^	−0.0224
	[−0.0373,0.0580]	[−0.2183,−0.1018]	[−0.0604,0.0347]	[−0.0609,0.1073]	[−0.3630,−0.1680]	[−0.1063,0.0614]
Marriage	0.2294^***^	−0.1018^***^	0.1848^***^	0.4123^***^	−0.1639^***^	0.3225^***^
	[0.1724,0.2865]	[−0.1702,−0.0334]	[0.1274,0.2423]	[0.3112,0.5134]	[−0.2772,−0.0507]	[0.2196,0.4255]
Primary school	−0.0165	0.0841^**^	−0.0764^**^	−0.0266	0.1474^**^	−0.1291^**^
	[−0.0750,0.0419]	[0.0136,0.1546]	[−0.1353,−0.0176]	[−0.1290,0.0758]	[0.0304,0.2644]	[−0.2351,−0.0230]
Middle school	−0.0052	0.1295^***^	−0.0241	0.0058	0.2256^***^	−0.0177
	[−0.0725,0.0621]	[0.0488,0.2103]	[−0.0915,0.0434]	[−0.1129,0.1246]	[0.0914,0.3598]	[−0.1377,0.1022]
High school & above	0.2088^***^	−0.0156	0.1203^***^	0.3910^***^	−0.0168	0.2161^***^
	[0.1274,0.2903]	[−0.1159,0.0846]	[0.0394,0.2013]	[0.2482,0.5339]	[−0.1848,0.1512]	[0.0744,0.3578]
Residence	−0.0332	−0.1089^***^	0.0761^***^	−0.0566	−0.1835^***^	0.1579^***^
	[−0.0789,0.0126]	[−0.1644,−0.0534]	[0.0301,0.1221]	[−0.1378,0.0246]	[−0.2761,−0.0909]	[0.0763,0.2395]
Single chronic disease	−0.5750^***^	0.5558^***^	−0.4559^***^	−1.0573^***^	1.0068^***^	−0.8242^***^
	[−0.6374,−0.5127]	[0.4717,0.6399]	[−0.5171,−0.3947]	[−1.1713,−0.9433]	[0.8512,1.1623]	[−0.9352,−0.7133]
Multiple chronic diseases	−0.9012^***^	1.1467^***^	−0.5310^***^	−1.5722^***^	1.9801^***^	−0.9193^***^
	[−0.9599,−0.8426]	[1.0682,1.2252]	[−0.5880,−0.4740]	[−1.6793,-1.4652]	[1.8338,2.1265]	[−1.0223,−0.8164]
Smoking	0.0624^**^	0.0291	0.0218	0.1152^**^	0.0345	0.0420
	[0.0098,0.1150]	[−0.0366,0.0949]	[−0.0316,0.0752]	[0.0227,0.2078]	[−0.0762,0.1453]	[−0.0525,0.1365]
Internet use	0.3148^***^	0.0029	0.2728^***^	0.5764^***^	0.0041	0.4861^***^
	[0.2649,0.3646]	[−0.0568,0.0626]	[0.2216,0.3239]	[0.4875,0.6653]	[−0.0951,0.1033]	[0.3942,0.5779]
Physical exercise	−0.0104	0.0361	−0.0056	−0.0202	0.0586	−0.0259
	[−0.0508,0.0300]	[−0.0149,0.0870]	[−0.0471,0.0359]	[−0.0914,0.0510]	[−0.0261,0.1433]	[−0.0989,0.0470]
Personal annual income (log)	0.0379^***^	0.0258^***^	0.0447^***^	0.0703^***^	0.0471^***^	0.0738^***^
	[0.0276,0.0483]	[0.0132,0.0385]	[0.0344,0.0549]	[0.0518,0.0889]	[0.0255,0.0687]	[0.0554,0.0922]
cut1	−2.5188^***^	1.3735^***^	−2.2604^***^	−4.8846^***^	2.3973^***^	−4.7223^***^
	[−2.6694,-2.3681]	[1.2025,1.5445]	[−2.4162,-2.1046]	[−5.1913,-4.5778]	[2.0971,2.6974]	[−5.0641,-4.3804]
cut2	−1.4626^***^		−1.4051^***^	−2.4978^***^		−2.5906^***^
	[−1.5947,-1.3305]		[−1.5394,-1.2707]	[−2.7348,-2.2608]		[−2.8372,-2.3440]
cut3	−0.1274^*^		−0.2142^***^	−0.1548		−0.3458^***^
	[−0.2577,0.0030]		[−0.3452,−0.0832]	[−0.3881,0.0784]		[−0.5809,−0.1106]
cut4	1.5282^***^		1.4369^***^	2.7186^***^		2.4244^***^
	[1.3940,1.6625]		[1.3045,1.5692]	[2.4769,2.9603]		[2.1861,2.6627]
*N*	11,455	11,455	11,455	11,455	11,455	11,455
*R* ^2^						
adj. *R*^2^						

95% confidence intervals in brackets. ^*^p < 0.1, ^**^p < 0.05, ^***^p < 0.01.

#### 3.3.2 Alternative health measures

To further verify the stability of our findings, we employed two alternative health indicators. Age-comparative self-rated health captures whether respondents perceive their health as better than, similar to, or worse than their age peers, dichotomized as 1 (better than or same as peers) and 0 (worse than peers). Health change over the past year assesses recent health trajectory, comparing current health to 1 year ago, dichotomized as 1 (better or stable) and 0 (deteriorated). Table 4 presents estimation results across OLS, Oprobit, and Ologit specifications. FDCS participation remains significantly associated with better health relative to peers across all model specifications (OLS: *β* = 0.0316, *p* < 0.001; Oprobit: *β* = 0.1878, *p* < 0.001; Ologit: *β* = 0.3680, *p* < 0.001), providing additional evidence for the robustness of our main association patterns. For health changes compared to the previous year, the association is marginally significant in the OLS model (*β* = 0.0151, *p* < 0.01) and directionally consistent though not statistically significant in the ordered models.

**Table 4:** Robustness check: Substituting alternative health outcome measures.

Variables	OLS model		Oprobit model		Ologit model	
	ASRH	Health change	ASRH	Health change	ASRH	Health change
FCDS	0.0316^***^	0.0151^*^	0.1878^***^	0.0542	0.3680^***^	0.1051
	[0.0190,0.0442]	[−0.0001,0.0303]	[0.0947,0.2809]	[−0.0242,0.1326]	[0.1793,0.5567]	[−0.0413,0.2516]
Oldest-old	−0.0105^*^	−0.0231^***^	−0.0767^*^	−0.1259^***^	−0.1494^*^	−0.2434^***^
	[−0.0215,0.0004]	[−0.0358,−0.0104]	[−0.1540,0.0007]	[−0.1951,−0.0566]	[−0.3035,0.0046]	[−0.3747,−0.1121]
Gender	0.0031	0.0179^**^	0.0098	0.0854^**^	0.0275	0.1642^**^
	[−0.0090,0.0152]	[0.0039,0.0319]	[−0.0701,0.0897]	[0.0129,0.1580]	[−0.1303,0.1853]	[0.0267,0.3016]
Marriage	0.0304^***^	0.0510^***^	0.1641^***^	0.2130^***^	0.2928^***^	0.3729^***^
	[0.0137,0.0471]	[0.0318,0.0702]	[0.0781,0.2502]	[0.1346,0.2915]	[0.1301,0.4554]	[0.2311,0.5146]
Primary school	0.0164^*^	0.0279^***^	0.0750^*^	0.1240^***^	0.1478^*^	0.2118^***^
	[−0.0001,0.0329]	[0.0091,0.0467]	[−0.0137,0.1637]	[0.0425,0.2054]	[−0.0207,0.3163]	[0.0638,0.3598]
Middle school	0.0077	0.0143	0.0273	0.0560	0.0358	0.0773
	[−0.0103,0.0257]	[−0.0065,0.0350]	[−0.0772,0.1319]	[−0.0389,0.1509]	[−0.1657,0.2372]	[−0.0967,0.2512]
High school & above	0.0242^**^	0.0392^***^	0.2080^***^	0.2149^***^	0.3931^***^	0.3849^***^
	[0.0045,0.0439]	[0.0163,0.0621]	[0.0655,0.3504]	[0.0919,0.3379]	[0.1003,0.6858]	[0.1497,0.6200]
Residence	−0.0019	−0.0060	−0.0174	−0.0360	−0.0390	−0.0617
	[−0.0137,0.0099]	[−0.0194,0.0074]	[−0.0926,0.0578]	[−0.1032,0.0313]	[−0.1853,0.1074]	[−0.1865,0.0630]
Single chronic disease	−0.0362^***^	−0.0430^***^	−0.4033^***^	−0.3348^***^	−0.8869^***^	−0.6921^***^
	[−0.0475,−0.0249]	[−0.0568,−0.0293]	[−0.5274,−0.2792]	[−0.4422,−0.2274]	[−1.1656,−0.6081]	[−0.9175,−0.4666]
Multiple chronic diseases	−0.0939^***^	−0.1279^***^	−0.7474^***^	−0.7404^***^	−1.5435^***^	−1.4356^***^
	[−0.1058,−0.0820]	[−0.1422,−0.1136]	[−0.8630,−0.6318]	[−0.8392,−0.6415]	[−1.8061,-1.2810]	[−1.6444,-1.2269]
Smoking	0.0020	−0.0131	0.0047	−0.0719^*^	0.0122	−0.1359^*^
	[−0.0115,0.0155]	[−0.0289,0.0026]	[−0.0847,0.0941]	[−0.1527,0.0089]	[−0.1652,0.1895]	[−0.2892,0.0174]
Internet use	0.0432^***^	0.0418^***^	0.2720^***^	0.2113^***^	0.5258^***^	0.3808^***^
	[0.0300,0.0564]	[0.0269,0.0566]	[0.1887,0.3554]	[0.1374,0.2853]	[0.3574,0.6942]	[0.2416,0.5200]
Physical exercise	0.0166^***^	0.0308^***^	0.1056^***^	0.1517^***^	0.1963^***^	0.2919^***^
	[0.0059,0.0273]	[0.0184,0.0431]	[0.0359,0.1753]	[0.0884,0.2151]	[0.0588,0.3339]	[0.1730,0.4108]
Personal annual income (log)	0.0023^*^	−0.0027^*^	0.0271^***^	−0.0098	0.0479^**^	−0.0218
	[−0.0001,0.0046]	[−0.0055,0.0002]	[0.0084,0.0458]	[−0.0254,0.0059]	[0.0100,0.0859]	[−0.0513,0.0078]
_cons	0.8756^***^	0.8853^***^				
	[0.8443,0.9069]	[0.8477,0.9229]				
cut1			−1.2722^***^	−1.3904^***^	−2.3685^***^	−2.5466^***^
			[−1.5066,-1.0378]	[−1.5979,-1.1829]	[−2.8546,-1.8823]	[−2.9512,-2.1420]
*N*	11,455	11,455	11,455	11,455	11,455	11,455
*R* ^2^	0.0372	0.0463				
adj. *R*^2^	0.0360	0.0452				

95% confidence intervals in brackets. *p < 0.1, **p < 0.05, ***p < 0.01.

#### 3.3.3 Propensity score matching

**Figure 2:**
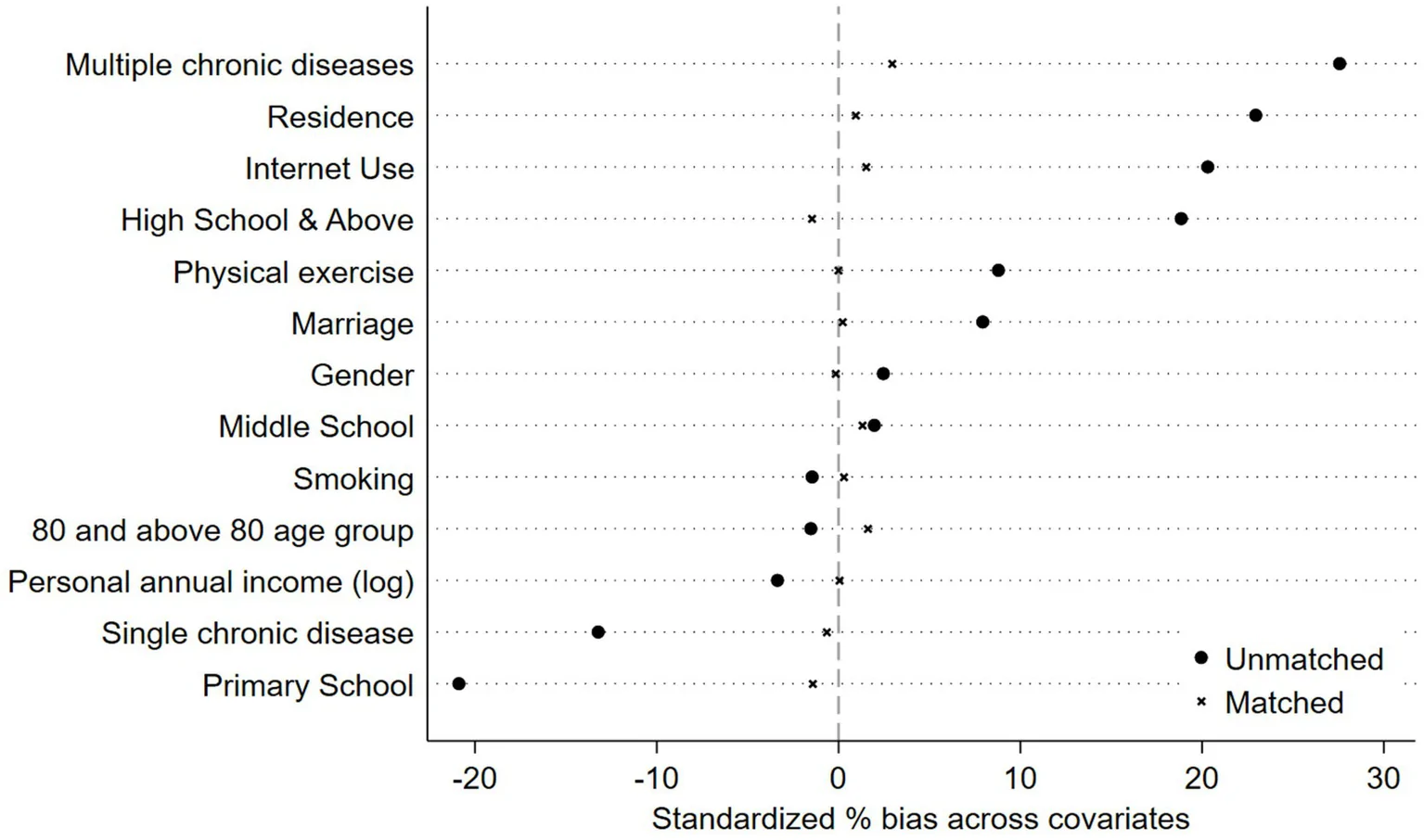
Covariate balance before and after matching.

**Figure 3:**
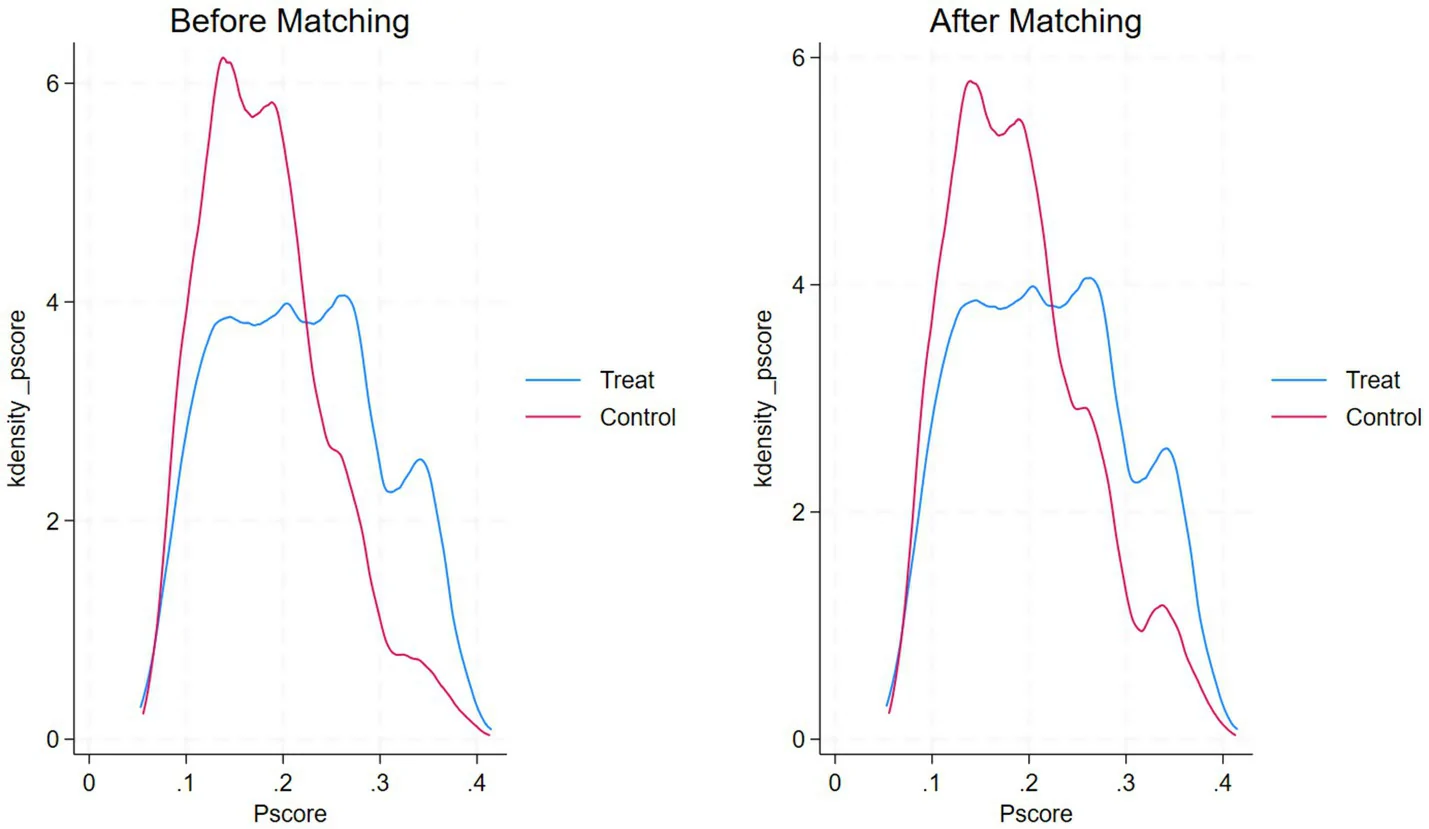
Kernel density plots of propensity scores for FDCS participants and non-participants.

PSM was implemented as a covariate-balance adjustment tool for observable selection into FDCS participation. Balance diagnostics confirmed successful covariate balancing across all four matching algorithms. Figure 2 presents a Love plot with detailed balance statistics (Table 5) comparing standardized bias for all covariates before and after matching. Before matching, nine out of 13 covariates exhibited significant differences between participants and non-participants (*p* < 0.05), with standardized bias exceeding 20% for several variables including multiple chronic diseases (27.6%), residence (23.0%), internet use (20.3%), and primary school education (20.9% in absolute value). After matching, post-matching standardized differences fell well below the 10% threshold for all variables, with the maximum bias of 2.9% observed for multiple chronic diseases and most variables achieving bias below 2%; no covariate showed significant differences (all *p*-values > 0.05), variance ratios ranged from 0.74 to 1.07 within acceptable bounds, and bias reduction percentages exceeded 80% for all variables. Figure 3 displays kernel density plots of propensity scores before and after matching, revealing substantial overlap in post-matching distributions and validating the common support assumption. As reported in Table 6, matched mean differences among FDCS participants remained consistently positive and statistically significant across all four matching algorithms and all three health outcomes. For self-rated health, matched differences ranged from 0.0392 (*p* < 0.10) under Mahalanobis matching to 0.0630 (*p* < 0.001) under k-nearest neighbor matching. FDCS participation showed robust associations with lower reported physical discomfort, with matched differences ranging from −0.0743 to −0.0785 (all *p* < 0.001), and greater life satisfaction, with matched differences between 0.0876 and 0.0953 (all *p* < 0.001) regardless of matching approach employed. The convergence of results across multiple matching specifications, combined with successful covariate balance achievement, strengthens confidence in the interpretation of associations between FDCS participation and health outcomes among older adults.

**Table 5:** Balance test results before and after PSM.

Variable	Unmatched/Matched	Mean treated	Mean control	%bias	%reduct|bias|	t	p > |t|	V(T)/V(C)
Primary school	Unmatched	0.2917	0.3902	−20.9000		−8.5800	0.0000	.
	Matched	0.2912	0.2979	−1.4000	93.2000	−0.4900	0.6270	.
Single chronic disease	Unmatched	0.2410	0.2996	−13.2000		−5.4300	0.0000	.
	Matched	0.2409	0.2438	−0.7000	95.0000	−0.2200	0.8230	.
Personal annual income (log)	Unmatched	10.1140	10.1850	−3.4000		−1.3200	0.1870	0.6200*
	Matched	10.1090	10.1080	0.0000	98.8000	0.0100	0.9890	0.7400*
Oldest-old	Unmatched	0.5876	0.5951	−1.5000		−0.6400	0.5230	.
	Matched	0.5875	0.5796	1.6000	−5.2000	0.5300	0.5990	.
Smoking	Unmatched	0.2677	0.2742	−1.5000		−0.6100	0.5430	.
	Matched	0.2677	0.2664	0.3000	80.4000	0.0900	0.9250	.
Middle school	Unmatched	0.2862	0.2773	2.0000		0.8300	0.4080	.
	Matched	0.2866	0.2807	1.3000	33.8000	0.4300	0.6690	.
Gender	Unmatched	0.5277	0.5154	2.5000		1.0300	0.3020	.
	Matched	0.5279	0.5288	−0.2000	93.1000	−0.0600	0.9560	.
Marriage	Unmatched	0.8544	0.8253	7.9000		3.2500	0.0010	.
	Matched	0.8546	0.8539	0.2000	97.3000	0.0700	0.9420	.
Physical exercise	Unmatched	0.4820	0.4382	8.8000		3.7000	0.0000	.
	Matched	0.4822	0.4823	0.0000	99.8000	−0.0100	0.9950	.
High school & above	Unmatched	0.2060	0.1354	18.8000		8.3500	0.0000	.
	Matched	0.2058	0.2113	−1.5000	92.2000	−0.4500	0.6540	.
Internet use	Unmatched	0.6152	0.5150	20.3000		8.4500	0.0000	.
	Matched	0.6156	0.6082	1.5000	92.6000	0.5000	0.6150	.
Residence	Unmatched	0.6009	0.4874	23.0000		9.5700	0.0000	.
	Matched	0.6013	0.5967	0.9000	95.9000	0.3100	0.7560	.
Multiple chronic diseases	Unmatched	0.6092	0.4731	27.6000		11.4800	0.0000	.
	Matched	0.6096	0.5951	2.9000	89.3000	0.9800	0.3290	.

*p < 0.1.

**Table 6:** PSM estimates of matched associations between FDCS participation and health outcomes among older adults.

Health of older adults	Matching methods			
	(1)	(2)	(3)	(4)
Panel A: self-reported health				
Treated	3.5981	3.5981	3.5981	3.5982
Controls	3.5351	3.5506	3.5465	3.5590
Matched association difference	0.0630^***^	0.0475^***^	0.0515^***^	0.0392^*^
S. E.	0.0212	0.0185	0.0180	0.0207
Panel B: physical discomfort				
Treated	0.2866	0.2866	0.2866	0.2866
Controls	0.3651	0.3619	0.3636	0.3609
Matched association difference	−0.0785^***^	−0.0753^***^	−0.0770^***^	−0.0743^***^
S. E.	0.0129	0.0114	0.0112	0.0131
Panel C: life satisfaction				
Treated	3.9206	3.9206	3.9206	3.9207
Controls	3.8253	3.8330	3.8310	3.8258
Matched association difference	0.0953^***^	0.0876^***^	0.0896^***^	0.0949^***^
S. E.	0.0203	0.0178	0.0174	0.0193

Column (1) shows estimates by k-nearest neighbor matching; Column (2) shows estimates by radius matching; Column (3) shows estimates by kernel matching; Column (4) shows estimates by Mahalanobis matching. Matched association differences are interpreted as covariate-balanced differences among FDCS participants, not as causal estimates. *p < 0.1, **p < 0.05, ***p < 0.01.

### 3.4 Heterogeneity analysis

Subgroup PSM models reveal a consistent dual-pattern heterogeneity across both residence and education dimensions (Tables 7, 8). Urban residents and higher-educated older adults show stronger associations with higher self-rated health, whereas rural residents and less-educated older adults show stronger associations with lower reported physical discomfort and higher life satisfaction. Supplementary Table S1 further separates education into illiterate, primary school, middle school, and high school or above categories. In that finer classification, self-rated-health associations are concentrated mainly among middle-school and high-school-or-above older adults, lower physical discomfort is most evident among illiterate and middle-school older adults, and life-satisfaction associations are positive across all four education levels. Supplementary Table S2 separates non-migrant rural and urban older adults from rural-to-urban and urban-to-rural migrants. The non-migrant rural and urban contrast is broadly consistent with the main binary residence results, whereas the two migrant subgroups show smaller, less precise, and more variable association patterns. These subgroup differences are descriptive association patterns after covariate balance adjustment and should not be read as evidence of tested mechanisms.

**Table 7:** Heterogeneity analysis by residence (Rural/Urban).

Health of older adults	Matching methods							
	(1)	(2)	(3)	(4)	(1)	(2)	(3)	(4)
	Rural older adults (N_T_ = 866; NC = 4,760)				Urban older adults (N_T_ = 1,304; NC = 4,525)			
Panel A: self-reported health								
Treated	3.5249	3.5249	3.5249	3.5242	3.6472	3.6472	3.6472	3.6472
Controls	3.5135	3.5177	3.5229	3.5349	3.5396	3.5432	3.5396	3.5745
Matched association difference	0.0114	0.0071	0.0020	−0.0107	0.1076^***^	0.1040^***^	0.1076^***^	0.0727^**^
S. E.	0.0328	0.0292	0.0288	0.0321	0.0287	0.0244	0.0240	0.0270
Panel B: physical discomfort								
Treated	0.2393	0.2393	0.2393	0.2402	0.3174	0.3174	0.3174	0.3174
Controls	0.3314	0.3495	0.3486	0.3363	0.3831	0.3761	0.3820	0.3744
Matched association difference	−0.0922^***^	−0.1102^***^	−0.1093^***^	−0.0961^***^	−0.0657^***^	−0.0587^***^	−0.0646^***^	−0.0569^***^
S. E.	0.0185	0.0164	0.0162	0.0179	0.0179	0.0158	0.0155	0.0181
Panel C: life satisfaction								
Treated	3.8485	3.8485	3.8485	3.8475	3.9693	3.9693	3.9693	3.9693
Controls	3.7532	3.7414	3.7403	3.7494	3.8686	3.8781	3.8745	3.8732
Matched association difference	0.0952^***^	0.1071^***^	0.1082^***^	0.0981^***^	0.1007^***^	0.0912^***^	0.0948^***^	0.0961^***^
S. E.	0.0317	0.0283	0.0280	0.0308	0.0273	0.0232	0.0228	0.0249

*p < 0.1, **p < 0.05, ***p < 0.01.

**Table 8:** Heterogeneity analysis by education level.

Health of older adults	Matching methods							
	(1)	(2)	(3)	(4)	(1)	(2)	(3)	(4)
	Primary school and below (N_T_ = 1,102; NC = 5,453)				Middle school and above (N_T_ = 1,068; NC = 3,832)			
Panel A: self-reported health								
Treated	3.5113	3.5113	3.5113	3.5117	3.6872	3.6872	3.6872	3.6872
Controls	3.4807	3.4745	3.4734	3.5170	3.5940	3.5925	3.6060	3.6191
Matched association difference	0.0306	0.0368	0.0379	−0.0052	0.0932^***^	0.0947^***^	0.0812^***^	0.0681^**^
S. E.	0.0287	0.0254	0.0253	0.0274	0.0326	0.0265	0.0259	0.0304
Panel B: physical discomfort								
Treated	0.2752	0.2752	0.2752	0.2749	0.2986	0.2986	0.2986	0.2986
Controls	0.3711	0.3626	0.3561	0.3457	0.3825	0.3827	0.3804	0.3625
Matched association difference	−0.0959^***^	−0.0874^***^	−0.0809^***^	−0.0707^***^	−0.0838^***^	−0.0840^***^	−0.0817^***^	−0.0639^***^
S. E.	0.0170	0.0150	0.0149	0.0165	0.0202	0.0171	0.0169	0.0215
Panel C: life satisfaction								
Treated	3.8583	3.8583	3.8583	3.8584	3.9850	3.9850	3.9850	3.9850
Controls	3.7356	3.7425	3.7394	3.7617	3.8602	3.8930	3.9065	3.8810
Matched association difference	0.1226^***^	0.1157^***^	0.1188^***^	0.0966^***^	0.1247^***^	0.0919^***^	0.0784^***^	0.1039^***^
S. E.	0.0281	0.0250	0.0249	0.0280	0.0304	0.0249	0.0244	0.0282

*p < 0.1, **p < 0.05, ***p < 0.01.

## 4 Discussion

This cross-sectional observational study shows that reported family doctor contract service (FDCS) participation is significantly associated with more favorable health outcomes across multiple dimensions among Chinese older adults. Using nationally representative data from the 2023 China Longitudinal Aging Social Survey and applying regression adjustment, ordered response models, and propensity score matching as a covariate-balance tool, we found that FDCS participation was consistently associated with higher self-rated health, lower reported physical discomfort, and greater life satisfaction across model specifications. The most consistent associations were observed for physical discomfort and life satisfaction. These associations vary systematically across subgroups: rural and less-educated older adults showed stronger associations with lower physical discomfort and higher life satisfaction, whereas urban and more educated older adults showed stronger associations with self-rated health. We interpret this heterogeneity as a theory-informed description of observed association differences, not as direct evidence of mechanisms.

Our findings align with and extend established evidence suggesting that primary healthcare strengthening is associated with more favorable health outcomes among older populations. Previous research has documented positive associations between FDCS participation and clinical indicators such as blood pressure control and medication adherence among patients with hypertension and diabetes (35, 36). Our study builds upon this foundation by showing that FDCS participation is associated not only with clinical biomarkers but also with multidimensional health outcomes including subjective health perceptions, physical symptom experiences, and overall life satisfaction. This comprehensive assessment suggests that favorable associations with FDCS participation may extend beyond disease management to broader dimensions of well-being that are central to healthy aging. However, our findings diverge from prior research in an important respect. While existing studies predominantly report average associations or simple binary assessments of policy performance (25, 37, 38), our research shows systematic heterogeneity patterns across sociodemographic dimensions and health outcome types. We employ a multidimensional health framework that simultaneously assesses cognitive (self-rated health), perceptual (physical discomfort), and affective (life satisfaction) dimensions, enabling identification of differential associations across health domains.

To interpret these heterogeneous patterns, we use healthcare accessibility theory and health literacy frameworks only as background. Among rural and less-educated older adults, the stronger associations with lower physical discomfort and higher life satisfaction may be interpreted as an accessibility-oriented pattern, because these groups often face greater barriers to routine care. These mechanisms are not directly observed in this study. The data support subgroup differences in adjusted associations, not the specific pathway, and future research should measure service content, care access, and symptom management directly.

For urban and better-educated older adults, the stronger association with self-rated health may be interpreted as a health-management-oriented pattern. These mechanisms are not directly observed in this study. What our data establish is that associations between FDCS participation and the three outcomes differ systematically across residence and education subgroups, whereas health literacy, preventive behavior, care coordination, and service content are not measured here. Future research should test these mediators directly and evaluate the cost and feasibility of differentiated service packages before large-scale adoption.

These findings carry cautious implications for FDCS policy and implementation. Because the present study identifies adjusted associations under an observational design rather than causal estimates, the following policy directions should be treated as evidence-informed suggestions and interpreted with appropriate caution. Their implementation would need to be further evaluated through experimental or longitudinal research. First, the dual-pattern heterogeneity suggests that one-size-fits-all service delivery may be poorly aligned with the observed needs of different older adult groups. Current FDCS implementation often applies standardized service packages uniformly across all participating older adults, but our findings suggest that differentiated service design may better align services with heterogeneous needs. For rural and less-educated older adults, an accessibility-oriented package could include scheduled village-clinic or home-based follow-up, routine pain screening, medication review, standardized guidance on essential analgesic use within primary-care prescribing authority, chronic disease monitoring, basic rehabilitation guidance, and referral pathways for persistent or severe pain. Where local workforce and regulatory conditions allow, contracted teams could integrate trained rehabilitation therapists or appropriate Traditional Chinese Medicine techniques with clear quality standards. For urban and higher-educated older adults, a knowledge-intensive package could include individualized health risk assessment reports, interactive health-literacy workshops, shared decision-making consultations, digital reminders, and structured self-management plans for chronic disease prevention. The feasibility of these packages depends on local conditions that vary across regions. Accessibility-oriented services presuppose that rural facilities have adequate human resources such as general practitioners and trained rehabilitation staff, and that prescribing authority for essential analgesics has been delegated to village and township clinicians, which is not yet uniform across localities. Knowledge-intensive services presuppose an organizing body able to host interactive health-literacy activities for urban older adults and mechanisms to sustain their participation over time. Because these conditions are unevenly met, the recommendations should be read as context-dependent options whose cost and feasibility require local assessment and further experimental or longitudinal evaluation before large-scale adoption. Second, quality assurance mechanisms should assess whether services reach groups with high observed needs. Performance evaluation systems that focus exclusively on enrollment rates or service provision counts risk overlooking whether services are aligned with the heterogeneous needs of diverse older adult populations. Quality metrics could assess whether disadvantaged groups receive accessibility-focused services associated with better daily health experiences, while advantaged groups receive knowledge-intensive services associated with health management capacity. This differentiated quality assessment framework may better capture whether FDCS implementation is aligned with both population health and health equity goals. Third, our findings have cautious implications for resource allocation. The stronger associations observed among disadvantaged populations suggest that targeted expansion of FDCS in underserved rural areas and among low-educated populations may be associated with favorable subjective well-being outcomes. However, this should not imply neglecting advantaged groups, for whom FDCS participation is also associated with favorable outcomes in the observed subgroup patterns. Rather, resource allocation could be coupled with service differentiation to ensure that all older adults receive services matched to their needs and capabilities. This nuanced approach moves beyond simplistic targeting versus universal coverage debates toward a more cautious understanding of how differentiated universal services may support more equitable service design.

Several limitations warrant acknowledgment. First, the cross-sectional nature of our data precludes causal interpretation. PSM addresses imbalance in observed covariates (39) but cannot eliminate residual confounding from unobservable factors such as health motivation, risk preferences, and trust in primary care. These unmeasured factors may bias the estimated associations, and the direction of any such bias is uncertain. Because behavioral variables such as physical exercise and internet use are measured at the same time as FDCS participation, they may act partly as mediators rather than as pure confounders, so that adjusting for them could absorb part of any association with FDCS. We had previously described the resulting estimates as conservative lower bounds, yet this presumes that any mediated component shares the sign of the direct association. This need not hold. FDCS participation might be associated with greater physical activity and better health, but greater activity could also carry offsetting risks such as injury, so the net mediated contribution and the direction of any over-adjustment are uncertain in both sign and magnitude. We were unable to apply instrumental-variable methods because the CLASS data contain no variable that plausibly satisfies the exclusion restriction. We therefore interpret the adjusted estimates as conditional associations and no longer describe them as definitively conservative, and we acknowledge the possibility of over-adjustment in either direction. Future research with longitudinal designs and richer measures of these constructs would be needed to evaluate temporality and mechanisms. Second, our binary FDCS participation measure does not differentiate service intensity or specific service components, potentially obscuring variation in service quality and quantity. China’s family doctor contracting program varies widely across regions in service content, intensity, and quality, and the well-documented “signed but not served” phenomenon means that nominal participants range from those holding only a registered contract to those receiving regular and substantive care. The data do not contain measures of service frequency, duration, continuity, provider contact, or quality, so we cannot estimate a dose–response relationship, stratify by service intensity, or correct for this misclassification. If the misclassification is largely non-differential with respect to the outcomes, it would tend to attenuate the estimated associations toward zero, in which case the associations among older adults who receive substantive services may be larger than those reported here under that interpretation. The available data do not, however, allow us to verify whether the misclassification is non-differential or to quantify its direction and magnitude. Future longitudinal studies should incorporate service frequency, duration, continuity, provider contact type, and perceived or objectively assessed service quality to determine whether more intensive and continuous FDCS engagement is associated with stronger health associations. Third, our health outcomes rely on self-reported assessments, which may be influenced by reporting biases. As noted in Section 2.2, SRH among Chinese older adults may be shaped by the common tendency to equate the absence of diagnosed illness with being healthy. We had previously argued that this would make our positive SRH associations a conservative lower bound, but on reflection this does not necessarily follow. A systematic reporting tendency introduces measurement error whose direction is not necessarily downward and could bias the SRH estimates in either direction, so we no longer describe these associations as uniformly conservative and we treat the direction of this potential bias as uncertain. We also considered using more objective health measures to corroborate the SRH findings, but the available indicators were not suitable as supplementary outcomes. The number of chronic conditions already enters our models as a need-factor covariate rather than as a dependent variable, and the activity of daily living items in the 2023 CLASS wave have substantial missingness that prevents their reliable use as alternative outcomes. We therefore rely on the convergence of findings across physical discomfort and life satisfaction, which are less susceptible to the illness-equivalence tendency, together with the alternative measures of age-comparative self-rated health and health change over the past year, as evidence that the broader pattern of positive associations is not an artifact of any single self-report measure. Fourth, the binary grouping strategy simplifies gradients within education and residence categories. To check that the dual-pattern heterogeneity is not an artifact of coarse categorization, we conducted supplementary subgroup analyses using a four-level education classification covering illiterate, primary school, middle school, and high school or above, together with a migration-sensitive residence classification that separates non-migrant rural and urban older adults from rural-to-urban and urban-to-rural migrants (Supplementary Tables S1, S2). These analyses are broadly consistent with the binary results but add two qualifications. First, the finer education results show that physical-discomfort associations are strong among both illiterate and middle-school older adults, so the education gradient should not be read as strictly monotonic. Second, the migration-sensitive residence results show that the two migrant subgroups have smaller samples and less precise estimates, so the main urban–rural contrast should be interpreted as a broad structural comparison rather than an exhaustive description of residence-related heterogeneity. Finally, while our nationally representative sample provides strong external validity for Chinese older adults, generalizability requires consideration of China’s specific policy context. Nonetheless, the broader theoretical insights regarding heterogeneous associations and the importance of matching services to population needs may have applicability to other aging societies implementing primary care reforms.

## 5 Conclusion

This study finds that reported FDCS participation is significantly associated with multiple health outcomes among older adults in China, including higher self-rated health, lower reported physical discomfort, and greater life satisfaction. These associations remained broadly consistent across OLS models, ordered probit and logit specifications, alternative health measures, and multiple propensity score matching approaches interpreted as covariate-balanced association differences. The heterogeneity analysis suggests that the observed association patterns are not uniform: lower physical discomfort and higher life satisfaction were more pronounced among rural residents and older adults with lower educational attainment, whereas higher self-rated health was more evident among urban and more educated older adults. These findings support a cautious, association-based interpretation that differentiated FDCS service design may be better aligned with the multidimensional health needs of diverse older adult subpopulations. Because the study is cross-sectional and observational, the results should be interpreted as adjusted associations rather than causal evidence about FDCS.

## Data Availability

The data used in the study are available from China Longitudinal Aging Social Survey (CLASS) dataset. Restrictions apply to the availability of these data, which were used under license for this study. Data are available at: http://class.ruc.edu.cn with the permission of CLASS team.
