# Supplementary material for: Strengthening primary care gatekeeping for healthy aging in China: family doctor contract service and multidimensional health outcomes among older adults
Source: Front Public Health. 2026 Aug 18;14:1875855. doi: 10.3389/fpubh.2026.1875855 (PMC13529558; doi:10.3389/fpubh.2026.1875855)
Supplement: Supplementary file 1 [file Table_1.docx]

Supplementary Material

# 1 Supplementary Tables

Supplementary Table S1. Four-Level Education Heterogeneity Analysis of Matched Associations Between FDCS Participation and Health Outcomes

| **Health of Older Adults** | **Matching Methods** | | | | | | | | | | | | | | | |
| --- | --- | --- | --- | --- | --- | --- | --- | --- | --- | --- | --- | --- | --- | --- | --- | --- |
|  | **(1)** | **(2)** | **(3)** | **(4)** | **(1)** | **(2)** | **(3)** | **(4)** | **(1)** | **(2)** | **(3)** | **(4)** | **(1)** | **(2)** | **(3)** | **(4)** |
|  | **Illiterate (NT=1830; NC=469)** | | | | **Primary school (NT=3623; NC=633)** | | | | **Middle school (NT=2575; NC=621)** | | | | **High school or above (NT=1257; NC=447)** | | | |
| **Panel A: self-reported health** | | | | | | | | | | | | | | | | |
| Treated | 3.5053 | 3.5053 | 3.5053 | 3.5053 | 3.5174 | 3.5174 | 3.5174 | 3.5165 | 3.6135 | 3.6135 | 3.6135 | 3.6135 | 3.7897 | 3.7892 | 3.7897 | 3.7897 |
| Controls | 3.4278 | 3.4311 | 3.4275 | 3.4275 | 3.5297 | 3.5148 | 3.5120 | 3.5501 | 3.5462 | 3.5236 | 3.5283 | 3.5237 | 3.7279 | 3.7044 | 3.6767 | 3.7511 |
| Matched association difference | 0.0775 | 0.0742* | 0.0778* | 0.0778 | -0.0123 | 0.0026 | 0.0054 | -0.0336 | 0.0673 | 0.0899*** | 0.0852** | 0.0898** | 0.0618 | 0.0848** | 0.1130*** | 0.0386 |
| S.E. | 0.0475 | 0.0428 | 0.0422 | 0.0478 | 0.0388 | 0.0322 | 0.0320 | 0.0353 | 0.0418 | 0.0344 | 0.0338 | 0.0399 | 0.0494 | 0.0429 | 0.0404 | 0.0445 |
| **Panel B: physical discomfort** | | | | | | | | | | | | | | | | |
| Treated | 0.2409 | 0.2409 | 0.2409 | 0.2409 | 0.2990 | 0.2990 | 0.2990 | 0.3001 | 0.3091 | 0.3091 | 0.3091 | 0.3091 | 0.2841 | 0.2847 | 0.2841 | 0.2841 |
| Controls | 0.3697 | 0.3522 | 0.3507 | 0.3358 | 0.3603 | 0.3555 | 0.3532 | 0.3645 | 0.4195 | 0.4025 | 0.4053 | 0.3969 | 0.3584 | 0.3704 | 0.3645 | 0.3366 |
| Matched association difference | -0.1288*** | -0.1113*** | -0.1098*** | -0.0949*** | -0.0613** | -0.0565*** | -0.0542*** | -0.0644*** | -0.1103*** | -0.0933*** | -0.0962*** | -0.0878*** | -0.0743** | -0.0857*** | -0.0804*** | -0.0525 |
| S.E. | 0.0266 | 0.0237 | 0.0233 | 0.0267 | 0.0239 | 0.0200 | 0.0199 | 0.0211 | 0.0262 | 0.0221 | 0.0217 | 0.0258 | 0.0305 | 0.0279 | 0.0268 | 0.0336 |
| **Panel C: life satisfaction** | | | | | | | | | | | | | | | | |
| Treated | 3.8486 | 3.8486 | 3.8486 | 3.8486 | 3.8655 | 3.8655 | 3.8655 | 3.8657 | 3.9436 | 3.9436 | 3.9436 | 3.9436 | 4.0425 | 4.0448 | 4.0425 | 4.0425 |
| Controls | 3.7532 | 3.7362 | 3.7360 | 3.7441 | 3.7839 | 3.7598 | 3.7497 | 3.7626 | 3.8624 | 3.8416 | 3.8531 | 3.8526 | 3.9575 | 3.9598 | 3.9685 | 3.9692 |
| Matched association difference | 0.0954** | 0.1124*** | 0.1126*** | 0.1045** | 0.0816** | 0.1057*** | 0.1158*** | 0.1031*** | 0.0812** | 0.1020*** | 0.0905*** | 0.0910** | 0.0850* | 0.0850** | 0.0740* | 0.0733* |
| S.E. | 0.0450 | 0.0403 | 0.0397 | 0.0435 | 0.0393 | 0.0329 | 0.0328 | 0.0363 | 0.0397 | 0.0325 | 0.0319 | 0.0365 | 0.0452 | 0.0399 | 0.0379 | 0.0387 |

Note. Each subgroup is matched separately. Columns (1) to (4) correspond to k-nearest neighbor matching with k=4, radius matching with caliper 0.01, kernel matching, and Mahalanobis distance matching. Treated and Controls are the matched group means of the outcome, the matched association difference is the difference between them, and S.E. is its standard error. Significance is denoted by *p<0.05, **p<0.01, and ***p<0.001. NT and NC denote the numbers of treated and control older adults in each subgroup.

Supplementary Table S2. Migration-Sensitive Residence Heterogeneity Analysis of Matched Associations Between FDCS Participation and Health Outcomes

| **Health of Older Adults** | **Matching Methods** | | | | | | | | | | | | | | | |
| --- | --- | --- | --- | --- | --- | --- | --- | --- | --- | --- | --- | --- | --- | --- | --- | --- |
|  | **(1)** | **(2)** | **(3)** | **(4)** | **(1)** | **(2)** | **(3)** | **(4)** | **(1)** | **(2)** | **(3)** | **(4)** | **(1)** | **(2)** | **(3)** | **(4)** |
|  | **Non-migrant rural older adults (NT=4215; NC=800)** | | | | **Rural-to-urban migrant older adults (NT=1319; NC=227)** | | | | **Urban-to-rural migrant older adults (NT=545; NC=66)** | | | | **Non-migrant urban older adults (NT=3206; NC=1077)** | | | |
| **Panel A: self-reported health** | | | | | | | | | | | | | | | | |
| Treated | 3.5106 | 3.5106 | 3.5106 | 3.5100 | 3.5330 | 3.5330 | 3.5330 | 3.5330 | 3.6969 | 3.6969 | 3.6969 | 3.6969 | 3.6713 | 3.6713 | 3.6713 | 3.6713 |
| Controls | 3.5011 | 3.5044 | 3.5115 | 3.5315 | 3.5185 | 3.5057 | 3.5070 | 3.4889 | 3.5909 | 3.6165 | 3.6181 | 3.6174 | 3.6022 | 3.5685 | 3.5712 | 3.6335 |
| Matched association difference | 0.0095 | 0.0062 | -0.0009 | -0.0215 | 0.0145 | 0.0273 | 0.0260 | 0.0441 | 0.1060 | 0.0804 | 0.0788 | 0.0795 | 0.0691** | 0.1028*** | 0.1001*** | 0.0378 |
| S.E. | 0.0342 | 0.0304 | 0.0301 | 0.0335 | 0.0653 | 0.0590 | 0.0579 | 0.0710 | 0.1143 | 0.1010 | 0.1007 | 0.1002 | 0.0321 | 0.0273 | 0.0269 | 0.0284 |
| **Panel B: physical discomfort** | | | | | | | | | | | | | | | | |
| Treated | 0.2352 | 0.2352 | 0.2352 | 0.2362 | 0.2422 | 0.2422 | 0.2422 | 0.2422 | 0.2878 | 0.2878 | 0.2878 | 0.2878 | 0.3333 | 0.3333 | 0.3333 | 0.3333 |
| Controls | 0.3384 | 0.3436 | 0.3427 | 0.3318 | 0.2671 | 0.3024 | 0.3029 | 0.3050 | 0.4542 | 0.4102 | 0.4126 | 0.4356 | 0.3879 | 0.3854 | 0.3910 | 0.3837 |
| Matched association difference | -0.1032*** | -0.1083*** | -0.1075*** | -0.0956*** | -0.0249 | -0.0602* | -0.0607* | -0.0628* | -0.1664** | -0.1223** | -0.1247** | -0.1477** | -0.0546*** | -0.0521*** | -0.0577*** | -0.0504** |
| S.E. | 0.0194 | 0.0169 | 0.0168 | 0.0185 | 0.0360 | 0.0321 | 0.0316 | 0.0366 | 0.0693 | 0.0608 | 0.0606 | 0.0669 | 0.0208 | 0.0183 | 0.0180 | 0.0211 |
| **Panel C: life satisfaction** | | | | | | | | | | | | | | | | |
| Treated | 3.8310 | 3.8310 | 3.8310 | 3.8300 | 3.8458 | 3.8458 | 3.8458 | 3.8458 | 4.0606 | 4.0606 | 4.0606 | 4.0606 | 3.9953 | 3.9953 | 3.9953 | 3.9953 |
| Controls | 3.7465 | 3.7203 | 3.7222 | 3.7447 | 3.7821 | 3.8090 | 3.8107 | 3.7962 | 3.8182 | 3.8999 | 3.9012 | 3.9015 | 3.9120 | 3.8945 | 3.8995 | 3.9254 |
| Matched association difference | 0.0845*** | 0.1107*** | 0.1088*** | 0.0853*** | 0.0637 | 0.0368 | 0.0351 | 0.0496 | 0.2424* | 0.1607 | 0.1594 | 0.1590 | 0.0833*** | 0.1008*** | 0.0958*** | 0.0699** |
| S.E. | 0.0328 | 0.0291 | 0.0289 | 0.0318 | 0.0623 | 0.0560 | 0.0552 | 0.0599 | 0.1297 | 0.1140 | 0.1137 | 0.1147 | 0.0301 | 0.0261 | 0.0257 | 0.0273 |

Note. Each subgroup is matched separately. Columns (1) to (4) correspond to k-nearest neighbor matching with k=4, radius matching with caliper 0.01, kernel matching, and Mahalanobis distance matching. Treated and Controls are the matched group means of the outcome, the matched association difference is the difference between them, and S.E. is its standard error. Significance is denoted by *p<0.05, **p<0.01, and ***p<0.001. NT and NC denote the numbers of treated and control older adults in each subgroup. Subgroups are defined by household registration and current residence. Non-migrant rural older adults hold agricultural registration and live in rural areas, rural-to-urban migrants hold agricultural registration but live in urban areas, urban-to-rural migrants hold non-agricultural registration but live in rural areas, and non-migrant urban older adults hold non-agricultural registration and live in urban areas.
